# High expression of TBRG4 in relation to unfavorable outcome and cell ferroptosis in hepatocellular carcinoma

**DOI:** 10.1186/s12885-024-11943-1

**Published:** 2024-02-12

**Authors:** Shanchun Tao, Di Cui, Huimin Cheng, Xiaofei Liu, Zhaobin Jiang, Hongwei Chen, Yong Gao

**Affiliations:** 1 Blood Transfusion Department, Fuyang Normal University Affiliated Second Hospital, Fuyang, Anhui 236000 China; 2 Fuyang Medical College, Fuyang Normal University, Fuyang, Anhui 236037 China; 3 School of Biology and Food Engineering, Fuyang Normal University, Fuyang, Anhui 236037 China; 4 Department of Clinical Laboratory, Fuyang Second People’s Hospital, Fuyang Infectious Disease Clinical College, Anhui Medical University, Fuyang, Anhui 236015 China

**Keywords:** TBRG4, Hepatocellular carcinoma, Proliferation, Ferroptosis

## Abstract

**Background:**

Hepatocellular carcinoma (HCC) is the most common type of malignant liver tumor with poor prognosis. In this study, we investigated the expression of transforming growth factor beta regulator 4 (TBRG4) in HCC and its effects on the proliferation, invasion, and metastasis of HCC cells, and analyzed the possible molecular mechanisms.

**Method:**

Downloading the expression and clinical information of HCC samples in the TCGA database, analyzing the expression differences of TBRG4 by bioinformatics methods, analyzing the clinical relevance and prognostic significance. Performing GO, KEGG and GSEA enrichment analysis on the TBRG4-related gene set in patient HCC tissues. Applying cell counting, scratch test and Transwell experiment to study the biological function of TBRG4 in HCC. Mitochondrial membrane potential, apoptosis and ROS levels were evaluated to assess cell iron death. Western blot, RT-PCR, laser confocal microscopy and co-immunoprecipitation were used to detect and analyze the downstream signaling pathways and interacting molecules of TBRG4.

**Results:**

Bioinformatics analysis revealed that TBRG4 was abnormally highly expressed in HCC tumor tissues and was associated with poor prognosis and metastasis in HCC patients. GO and KEGG functional enrichment analysis showed that TBRG4 was related to oxidative stress and NADH dehydrogenase (ubiquinone) activity. GSEA enrichment analysis showed that TBRG4 was associated with Beta catenin independent wnt signaling and B cell receptor. Functional experiments confirmed that knocking down TBRG4 could inhibit the proliferation, migration, and invasion of HCC cells. Mechanistically, TBRG4 inhibited the function of HCC cells through the DDX56/p-AKT/GSK3β signaling pathway. In addition, interference with TBRG4 expression could reduce the mitochondrial membrane potential and accumulate ROS in HCC cells, leading to increased ferroptosis. Co-IP analysis showed that TBRG4 specifically bound to Beclin1.

**Conclusion:**

TBRG4 is highly expressed in HCC tumor tissues and is associated with poor prognosis. It may regulate the proliferation, invasion, and metastasis of HCC cells through the DDX56/p-AKT/GSK3β signaling pathway. TBRG4 may interact with Beclin1 to regulate the ferroptosis of HCC cells.

**Supplementary Information:**

The online version contains supplementary material available at 10.1186/s12885-024-11943-1.

## Introduction

Liver cancer is ranked sixth globally in terms of tumor incidence rate and third in terms of mortality [1, 2]. Hepatocellular carcinoma (HCC) accounts for 90% of liver cancer cases [3]. Hepatitis B virus infection is the main risk factor for liver cancer, with chronic hepatitis B related liver cancer cases accounting for about 50% of the total cases [4]. With the improvement of living standards, nonalcoholic steatohepatitis (NASH), metabolic syndrome and diabetes are also gradually becoming important causes of liver cancer [5]. Although significant progress has been made in the diagnosis and treatment of HCC in recent years, the overall treatment effect is not satisfactory, especially in the treatment of mid to late stage, which still faces many challenges [6, 7]. Consequently, it is crucial to conduct in-depth research on HCC invasion and metastasis, explore the mechanisms underlying these processes, and identify new treatment targets to enhance the outcomes for mid to late-stage patients.

Transforming growth factor beta regulator 4 (TBRG4), also known as Fas activated serine/threonine kinase domain containing protein 4 (FASTKD4) [8]. Sequence analysis reveals that the protein encoded by TBRG4 may exert control over the cell cycle by stabilizing cell cycle regulatory proteins at the transcriptional level, facilitated by the presence of a leucine zipper motif [9]. The TBRG4 gene is located at 7q14-p13 [8], and its aberrant expression is closely associated with tumorigenesis. Studies have demonstrated a significant increase in TBRG4 expression in patients with head and neck squamous cell carcinoma and in the tumor tissues of multiple myeloma patients with extramedullary recurrence [10, 11]. Knockdown of TBRG4 significantly inhibited the growth and proliferation of breast cancer cell lines [12]. In addition, TBRG4 is abnormally overexpressed in osteosarcoma and esophageal squamous cell carcinoma, and can regulate cell proliferation and invasion through PI3K/AKT [13, 14]. However, the specific role of TBRG4 in hepatocellular carcinoma (HCC) remains unclear. This study aims to employ bioinformatics methods to analyze the clinical significance of TBRG4 expression in HCC. Molecular biology experimental techniques will also be used to investigate whether TBRG4 is involved in the biological behaviors of HCC, such as cell proliferation, invasion, and migration. These findings will provide an experimental foundation for the preliminary exploration of TBRG4 as a potential therapeutic target in HCC.

## Materials and methods

### Data collection

Download and organize RNAseq data for the STAR process of the TCGA-ALL (pan cancer) project from the TCGA database (https://portal.gdc.cancer.gov), including 371 cancer samples and 50 adjacent cancer samples. Convert PFKM format RNA seq data into TPM format and extract corresponding numbers to match adjacent and cancerous samples for parsing pan cancerous data expression. Then use the R software "limma" package for log2 conversion analysis [15]. In addition, clinical information HCC patients were obtained from TCGA. RNAseq data and clinical information of 240 liver cancer samples and 202 normal samples were obtained from ICGC database (https://dcc.icgc.org/releases/current/Projects). Clinical information and RNA data of GSE14520 were downloaded from GEO database (https://www.ncbi.nlm.nih.gov/gds).

### Analysis of TBRG4 expression level and clinical significance

Using R software to read the gene expression data and clinical data of HCC samples in the TCGA database, the "survival" package, "survivor" package, and "time ROC" package were used to draw the overall survival curve, ROC curve and COX regression analysis forest map, respectively. For Kaplan–Meier curves, *p*-values and hazard ratio (HR) with 95% confidence interval (CI) were generated by log-rank tests and univariate cox proportional hazards regression. Clinical correlation analysis selects appropriate statistical methods based on the characteristics of the data format ("stats" package and "car" package) and visualizes the data using "ggplot2" package.

### Nomogram related models and prognostic calibration curves

Using "survival package" for proportional risk hypothesis testing and Cox regression analysis, constructing nomogram related models and Calibration analysis using the "RMS" package for indicators with significant Univariate analysis prognosis.

### Screening and enrichment analysis of related genes

Using LinkedOmics (http://www.Thelinkedomics.Org) database was used to obtain genes positively and negatively correlated with TBRG4 expression with the threshold for |pearson correlation coefficient|> 0.6 and *p* -value < 0.01. The R package "org. Hs. eg. db" was used for ID conversion, and the package "cluster profile" was used for GO and KEGG enrichment analysis. The enriched gene categories include MF (Molecular Function), CC (Cellular Component), BP (Biological Process) and KEGG (Kyoto Encyclopedia of Genes and Genomes). For Gene set enrichment analysis (GSEA), we use "clusterProfiler" package for parsing. We ranked the TBRG4 related genes according to their correlation coefficients and obtained them from the Molecular Signatures Database (http://www.gsea-msigdb.org/gsea/downloads.jsp) downloaded the subset of c2.cp.kegg.v7.4.symbols.gmt to evaluate relevant pathways and molecular mechanisms. Based on the predetermined gene ranking to analysis, we set the minimum gene set to 5 and the maximum gene set to 5000, with 1000 resamples, *P* -value < 0.05 and FDR < 0.25 were considered statistically significant.

### Protein–protein interaction (PPI) network and immune infiltration analysis

Build a PPI network using the GeneMANIA database to predict the interaction proteins and related functions of TBRG4. Based on the ssGSEA algorithm provided in package "GSVA", the immune infiltration of TBRG4 was calculated using the markers of 24 immune cells provided in the study of Bindea, Gabriela, et al [16].

### Cell culture

The hepatoma cell lines LO2, Hep3B, SMC7721, HepG2 and Huh7 used in this experiment were provided by the Environmental Hormones and Reproductive Development Laboratory of Fuyang Normal University. LO2, HepG2, SMC7721 and Huh7 were cultured in DMEM medium containing 10% fetal bovine serum, 1% penicillin–streptomycin, while HepG2 was cultured in DMEM medium containing 10% fetal bovine serum, 1% penicillin–streptomycin and 1% sodium pyruvate in a cell incubator at 37 ℃ and 5% CO2. The cell culture process should follow the principle of sterility, and the medium should be changed on time. The above cell lines should be passaged at a ratio of 1:2.

### Migration and invasion assay

Inoculate the target cells and their control cells into upper chamber without Matrigel (for migration) or with Matrigel (for invasion) and add serum-free culture medium. Add DMEM culture medium containing 20% fetal bovine serum to the bottom of the chamber, incubate at 37 ℃ in a 5% CO2 incubator for 36 to 48 h, and fix the cells that migrated to the membrane. Use crystal violet with a concentration of 0.5% for cell staining, wash and float dry, and observe under a microscope.

### Cell scratch experiment

After 24 h of transfection, the cells were inoculated onto a 6-well plate. On the second day of cultivation, verify that the cell density meets the experimental requirements. Use a pipette gun to draw a line in a 6-well plate that is perpendicular to the bottom horizontal line. Gently clean the cells with PBS three times, remove the scratched cells, and add serum-free medium for further cultivation. Observe the healing of scratches at 0 and 24 h after marking.

### Clone formation experiment

Digest and passage the target cells and their control cells, prepare a cell suspension, count the cells, and inoculate 200 cells in a 6-well plate for 10–14 days in complete culture medium. After observing cell clones visible to the naked eye, discard the culture medium and wash the cells with PBS three times. Fix with methanol for 15 min, stain with crystal violet for 10 min, slowly wash off the dye solution with running water, and observe the formation of clones.

### Cell transfection

Take logarithmic growth phase cells, wait for the cell density to reach about 80%, and proceed with transfection according to the instructions. Using serum free medium, add Lipo8000 transfection reagent (Biyuntian, Shanghai, China) and plasmid, let stand for 10 min, slowly add to the medium and mix well. After 16 h, replace the medium. After 48 h, collect cells to test transfection efficiency.

### Immunohistochemistry

A total of 8 HCC tumor tissue with their adjacent non-cancerous lung tissues used in this study were obtained from the Second Affiliated Hospital of Fuyang Normal University and were approved by the hospital's ethics committee. The collected tissue samples were prepared and stored by paraffin embedding, then dewaxed, and sealed in a methanol solution. After tissue sectioning, antigen repair was performed using sodium citrate EDTA. Afterwards, the slices were incubated overnight with TBRG4 antibody (Proteintech, Wuhan, China), followed by incubation of the secondary antibody and staining. Images were obtained using a scanning microscope and analyzed.

### Immunofluorescence

Inoculate logarithmic growth phase cells into a confocal microscope culture dish, fix with paraformaldehyde for 10 min, and then block with goat serum for 1 h. Incubate the cells with TBRG4 and TOM20 antibodies overnight. After washing with PBS three times, incubate the fluorescent secondary antibody, and then add DAPI staining solution dropwise. Use laser confocal microscope to take photos and analyze.

### PCR and Western Blot

The RT-PCR and Western Blot experimental methods can be found in previous studies [17]. β-Actin is used as an internal control for RNA quantification standardization. Using primer sequences β-Actin (F: CTCGCCTTTGCCGATCC, R: GAATCCTTCTGACCCATGCC) and TBRG4 (F: CAGCTCACCTGGTAAAGCGAT, R: GGGAGTAGATGCTCGTTCCTTC). β-Actin, TBRG4, AKT, p-AKT, BCL2, GSK3β, ACSL4 and GPX4 antibodies are ordered from Proteintech (Wuhan, China), Beclin1 and TF antibodies are ordered from Biyuntian (Shanghai, China), and DDX56 is ordered from SANTA (St Louis, USA). All blots were cut prior to hybridization with antibodies. According to the required target proteins, the membrane was cut open and incubated in different boxes to eliminate the interference of some impurity bands. The original image of the blots used is included in Supplementary Fig. 2.

### Co-immunoprecommendation(Co-IP)

Collect logarithmic growth phase cells, add 1 mL RIPA lysate, centrifuge, add agarose beads and TBRG4 antibody, and shake overnight. Collect agarose beads, add SDS sampling buffer, and boil for 100 min. Staining gel with Fast Silver Stain Kit (Biyuntian, Shanghai, China) and use Western Blot to detect TBRG4 interacting molecules.

### Statistical analysis

Statistical analysis was performed using R-3.6.1, GraphPad Prism 8, and ImageJ software. Using independent sample t-test to compare normal distribution data between two groups. Survival analysis was conducted using the logarithmic rank (Mantel Cox) test. *P*-value less than 0.05 was considered statistically significant.

## Results

### Analysis of TBRG4 expression levels in HCC patients and pan-cancers

**Fig. 1:**
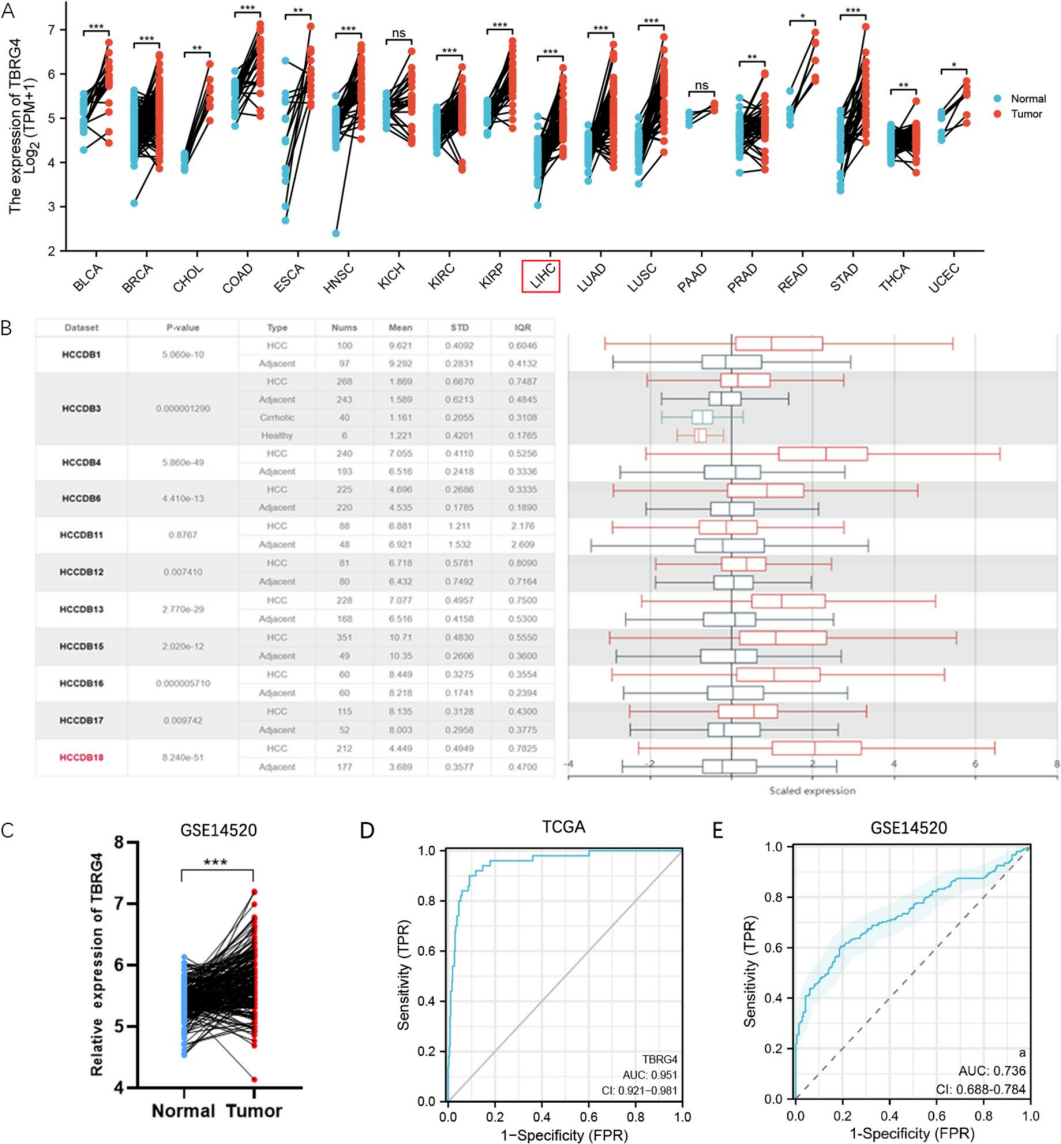
Analysis of TBRG4 expression levels in HCC patients and pan-cancers. **A** TBRG4 expression in paired samples in pan-cancer data of The Cancer Genome Atlas (TCGA). **B** Differential expression of TBRG4 in HCCDB. **C** ROC Analysis of TBRG4 in HCC Diagnosis. **D** Correlation analysis between the expression level of TBRG4 and the pathological stage of HCC patients. **E** Correlation analysis between the expression level of TBRG4 and the T stage of HCC patients. Data were shown as mean ± SD. *:* p* < 0.05, **: *p* < 0.01, ***: *p* < 0.001, ****: *p* < 0.0001

We first analyzed the paired sample expression of TBRG4 in the TCGA database. The results showed that TBRG4 showed abnormal elevation in 14 types of tumors, including HCC (Fig. 1A). In addition, we also used the HCC database for analysis and found that TBRG4 showed a significant increase in 10 datasets (Fig. 1B). Similarly, in the tumor cohorts of GSE14520 and ICGC, the expression level of TBRG4 also showed a significant increase (Fig. 1C and Supplemental Fig. 1A). ROC curve analysis shows that TBRG4 is a good diagnostic marker for HCC patients (Fig. 1D and 1E). In addition, we divided TCGA HCC patients into high expression and low expression groups based on the median expression of TBRG4 and analyzed the clinical characteristics of the two groups of patients (Table 1). The results showed that the abnormal high expression of TBRG4 is related to Histologic grade, Vascular invasion, Weight, BMI and AFP in HCC patients. In the ICGC patient’s cohort, the expression level of TBRG4 is positively correlated with the histologic grade of HCC (Supplemental Fig. 1B).

**Table 1:** HCC patient characteristics of HCC and TBRG4 expression correlated with clinical-pathological characteristics

Characteristics	Low expression of TBRG4	High expression of TBRG4	*p*value
N	187	187	
Pathologic T stage, n (%)			0.007
T1	107 (28.8%)	76 (20.5%)	
T2	41 (11.1%)	54 (14.6%)	
T3	32 (8.6%)	48 (12.9%)	
T4	4 (1.1%)	9 (2.4%)	
Pathologic N stage, n (%)			0.659
N0	124 (48.1%)	130 (50.4%)	
N1	1 (0.4%)	3 (1.2%)	
Pathologic M stage, n (%)			0.999
M0	127 (46.7%)	141 (51.8%)	
M1	2 (0.7%)	2 (0.7%)	
Pathologic stage, n (%)			0.003
Stage I	103 (29.4%)	70 (20%)	
Stage II	39 (11.1%)	48 (13.7%)	
Stage III	31 (8.9%)	54 (15.4%)	
Stage IV	3 (0.9%)	2 (0.6%)	
Tumor status, n (%)			0.009
Tumor free	114 (32.1%)	88 (24.8%)	
With tumor	65 (18.3%)	88 (24.8%)	
Histologic grade, n (%)			0.0001
G1	34 (9.2%)	21 (5.7%)	
G2	103 (27.9%)	75 (20.3%)	
G3	43 (11.7%)	81 (22%)	
G4	4 (1.1%)	8 (2.2%)	
Gender, n (%)			0.581
Female	58 (15.5%)	63 (16.8%)	
Male	129 (34.5%)	124 (33.2%)	
Race, n (%)			0.120
Asian	73 (20.2%)	87 (24%)	
Black or African American	6 (1.7%)	11 (3%)	
White	101 (27.9%)	84 (23.2%)	
Age, n (%)			0.957
< = 60	89 (23.9%)	88 (23.6%)	
> 60	98 (26.3%)	98 (26.3%)	
Weight, n (%)			0.000
< = 70	73 (21.1%)	111 (32.1%)	
> 70	99 (28.6%)	63 (18.2%)	
Height, n (%)			0.268
< 170	94 (27.6%)	107 (31.4%)	
> = 170	74 (21.7%)	66 (19.4%)	
BMI, n (%)			0.001
< = 25	73 (21.7%)	104 (30.9%)	
> 25	94 (27.9%)	66 (19.6%)	
Histological type, n (%)			0.117
Fibrolamellar carcinoma	3 (0.8%)	0 (0%)	
Hepatocellular carcinoma	182 (48.7%)	182 (48.7%)	
Hepatocholangiocarcinoma (mixed)	2 (0.5%)	5 (1.3%)	
Residual tumor, n (%)			0.904
R0	165 (47.8%)	162 (47%)	
R1	8 (2.3%)	9 (2.6%)	
R2	1 (0.3%)	0 (0%)	
AFP(ng/ml), n (%)			0.001
< = 400	122 (43.6%)	93 (33.2%)	
> 400	22 (7.9%)	43 (15.4%)	
Albumin(g/dl), n (%)			0.648
< 3.5	38 (12.7%)	31 (10.3%)	
> = 3.5	120 (40%)	111 (37%)	
Prothrombin time, n (%)			0.850
< = 4	110 (37%)	98 (33%)	
> 4	46 (15.5%)	43 (14.5%)	
Vascular invasion, n (%)			0.039
No	116 (36.5%)	92 (28.9%)	
Yes	48 (15.1%)	62 (19.5%)	
Child–Pugh grade, n (%)			0.495
A	114 (47.3%)	105 (43.6%)	
B	13 (5.4%)	8 (3.3%)	
C	1 (0.4%)	0 (0%)	
Adjacent hepatic tissue inflammation, n (%)			0.126
None	70 (29.5%)	48 (20.3%)	
Mild	49 (20.7%)	52 (21.9%)	
Severe	7 (3%)	11 (4.6%)	

Data were shown as mean ± SD. *p < 0.05, **p < 0.01, ***p < 0.001, ****p < 0.0001

### The impact of TBRG4 expression level on the prognosis of HCC patients

**Fig. 2:**
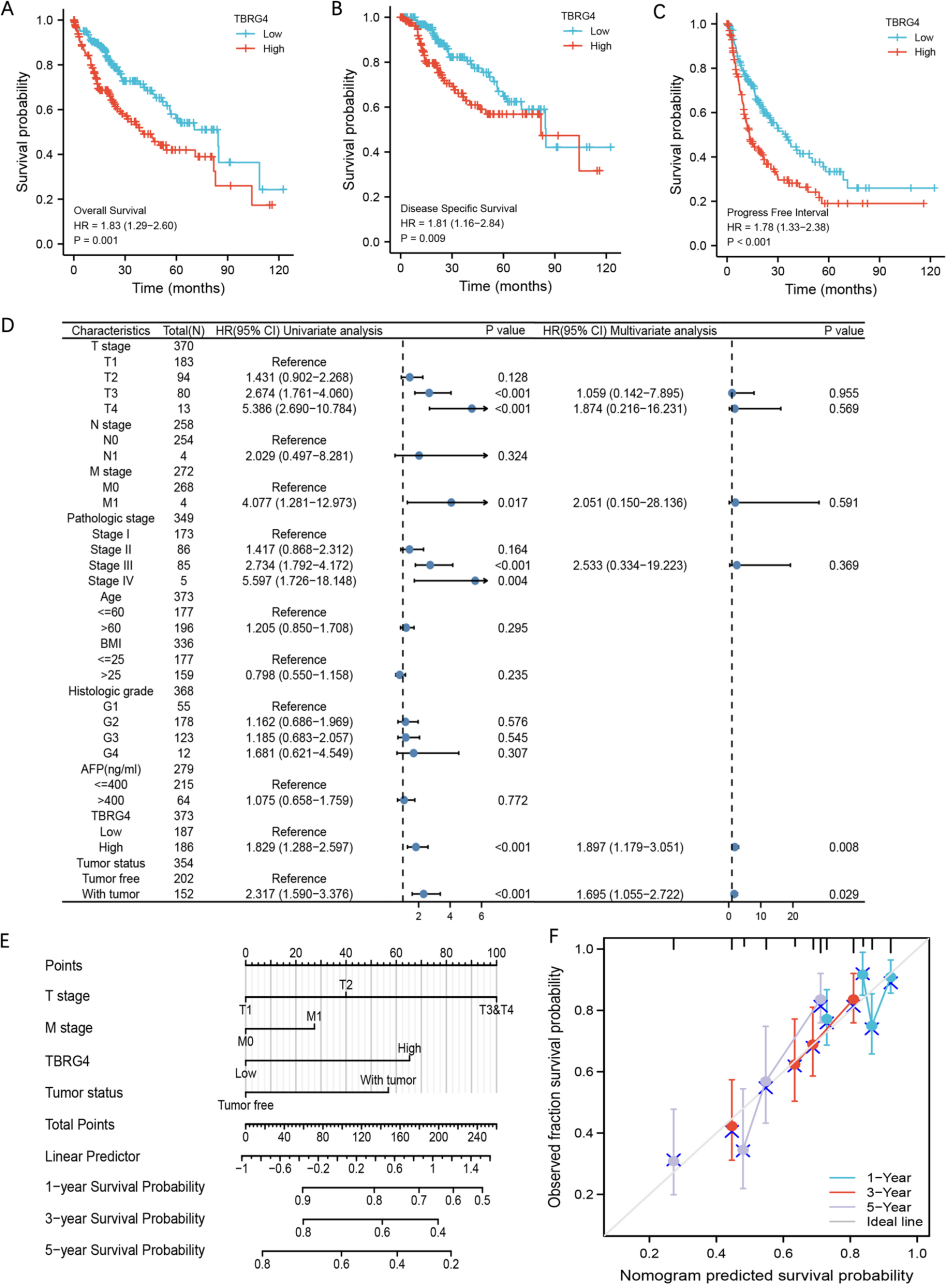
The impact of TBRG4 expression level on the prognosis of HCC patients. **A** Analysis of the correlation between TBRG4 expression and HCC patient OS. **B** Analysis of the correlation between TBRG4 expression and HCC patient DSS. **C** Analysis of the correlation between TBRG4 expression and HCC patient PFI. **D** Univariate and multivariate Cox analysis of TBRG4 expression and prognosis in HCC patients. **E** The nomogram of TBRG4 in the prognosis of AML. **F** Calibration analysis of TBRG4 in the prognosis of HCC

Prognostic analysis shows that HCC patients with high TBRG4 expression have poorer overall survival(OS), disease specific survival(DSS) and progress free interval(PFI), as depicted in Fig. 2A, B, and C. In the ICGC patient’s cohort, the high expression group of TBRG4 also showed poor prognosis (Supplemental Fig. 1C). AUC curve analysis indicates that TBRG4 can serve as a prognostic marker for ICGC patients (Supplemental Fig. 1D). Next, we conducted univariate and multivariate COX regression analysis using the clinical characteristics and TBRG4 expression levels of HCC patients (Fig. 2D). Univariate analysis showed that the prognosis of patients was related to the T/M staging, pathological grade, TBRG4 expression level, and tumor status. Multivariate analysis showed that only TBRG4 and tumor status were prognostic factors for HCC patients. Next, we use the “RMS” package to build nomogram related models and Calibration analysis (Fig. 2E and F) and found that TBRG4 can accurately predict the survival prognosis of hepatocellular carcinoma patients. These results suggest that TBRG4 may be one of the important prognostic factors for HCC patients.

### TBRG4 related molecules and enrichment analysis

**Fig. 3:**
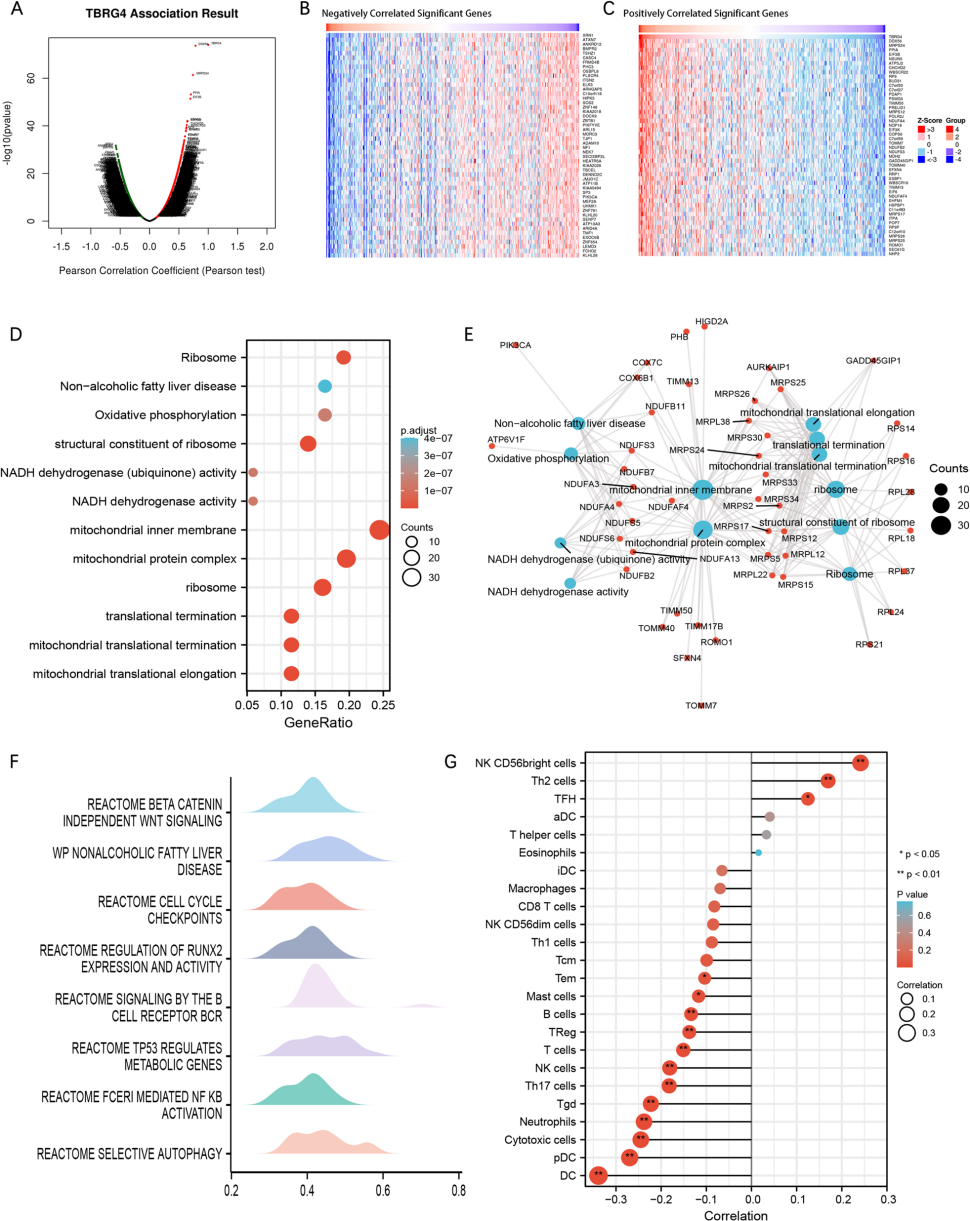
TBRG4 related molecules and enrichment analysis. **A** Pearson test was used to analyze the association of TBRG4 with genes expressed in HCC cohort. **B** Heat maps showing TOP 50 genes positively related to TBRG4. **C** Heat maps showing TOP 50 genes negatively related to TBRG4. **D** Pathways related to GO and KEGG enrichment analysis. **E** Visual network of GO and KEGG enrichment analysis. **F** GSEA analysis of the top ten enrichment pathways of Enrichment Mountain plots. **G** Correlation between expression of TBRG4 and immune infiltration

To investigate the potential biological function of TBRG4, we utilized the LinkedOmics database and performed a Spearman test to identify genes associated with TBRG4 expression in HCC patients. Our analysis revealed that TBRG4 expression was positively correlated with 4167 genes, while it was negatively correlated with 7145 genes (Fig. 3A). We further examined the top 50 genes that showed positive correlation (Fig. 3B) and negative correlation (Fig. 3C) with TBRG4. Additionally, we identified 168 genes that exhibited a correlation coefficient greater than 0.5, indicating co-expression with TBRG4. Conduct GO, KEGG and GSEA enrichment analysis on the TBRG4 related gene set, and screen the enrichment results according to FDR < 0.01(Fig. 3D and E). The enrichment analysis results of GO and KEGG indicate that TBRG4 may be related to Non-alcoholic fatty liver disease, Oxidative phosphorylation, structural constituent of ribosome, NADH dehydrogenase (ubiquinone) activity. The GSEA analysis results show that TBRG4 is associated with multiple HCC related signaling pathways, such as Beta catenin independent wnt signaling, nonalcoholic fatty liver disease, B cell receptor and TP53 regulates metabolic genes et at. In addition, we used GSVA packages to analyze the relationship between TBRG4 expression levels and immune infiltration in HCC. The results showed that TBRG4 was positively correlated with NK CD56bright cells, Th2 cells, TFH cells and negatively correlated with DC cells, cytotoxic cells, neutrophil cells. This means that TBRG4 may be an important marker of immune infiltration in HCC.

### PPI interaction network and drug sensitivity analysis

**Fig. 4:**
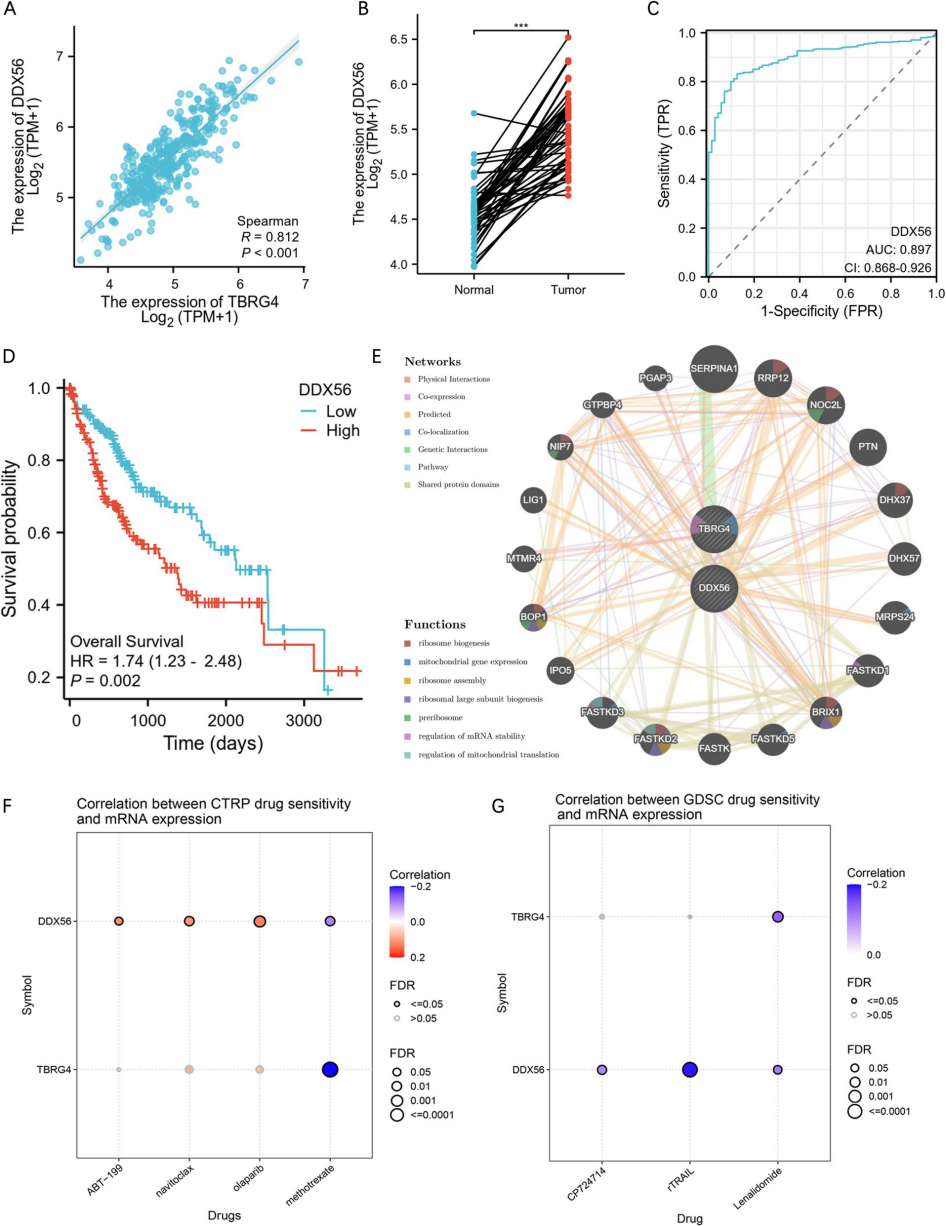
PPI Interaction Network and Drug Sensitivity Analysis. **A** Analysis of the expression correlation between TBRG4 and DDX56 in HCC. **B** Differential expression of DDX56 in HCC. **C** ROC Analysis of DDX56 in HCC Diagnosis. **D** The prognosis and expression of DDX56 in HCC. **E** PPI network of TBRG4 and DDX56 using GeneMANIA. **F** Correlation between CTRP drug sensitivity and mRNA expression. **G** Correlation between GDSC drug sensitivity and mRNA expression. Data were shown as mean ± SD. *:* p* < 0.05, **: *p* < 0.01, ***: *p* < 0.001, ****: *p* < 0.0001

During our molecular analysis concerning TBRG4, we observed a remarkably high correlation between TBRG4 and DEAD-Box 56 (DDX56), with a correlation coefficient of 0.812 (Fig. 4A). In the GSE14520 and ICGC cohort, DDX56 also showed a strong correlation with TBRG4 (Supplemental Fig. 1E and F). In paired sample analysis, DDX56 was significantly elevated in HCC tissue (Fig. 4B). The ROC curve and survival analysis showed that DDX56 is also an important factor in the diagnosis and prognosis of HCC patients (Fig. 4C and D). This strong correlation suggests a potential functional relationship or co-regulation between these two factors, which may be of significance in understanding their roles in biological processes. Further investigation into the interplay between TBRG4 and DDX56 is warranted to uncover the underlying mechanisms and implications for relevant pathways or functions. Therefore, we used the GeneMANIA database to construct a PPI interaction network for TBRG4 and DX56, with functions focused on ribosome biogenesis, mitochondrial gene expression and ribosome assembly et al. (Fig. 4E). Next, we used the GSCA database (http://bioinfo.life.hust.edu.cn/GSCA/#/drug) to analyze the drug sensitivity of TBRG4 and DDX56 (Fig. 4F and G). Drug names, Correlation, and FDR can be found in Supplementary Table 1. It was found that TBRG4 may be related to resistance to methotrexate and lenalidomide. For commonly used drugs in HCC, we found that TBRG4 may be associated with resistance to Vinblastine.

### TBRG4 exhibits abnormally high expression in HCC cell lines and tissue samples

**Fig. 5:**
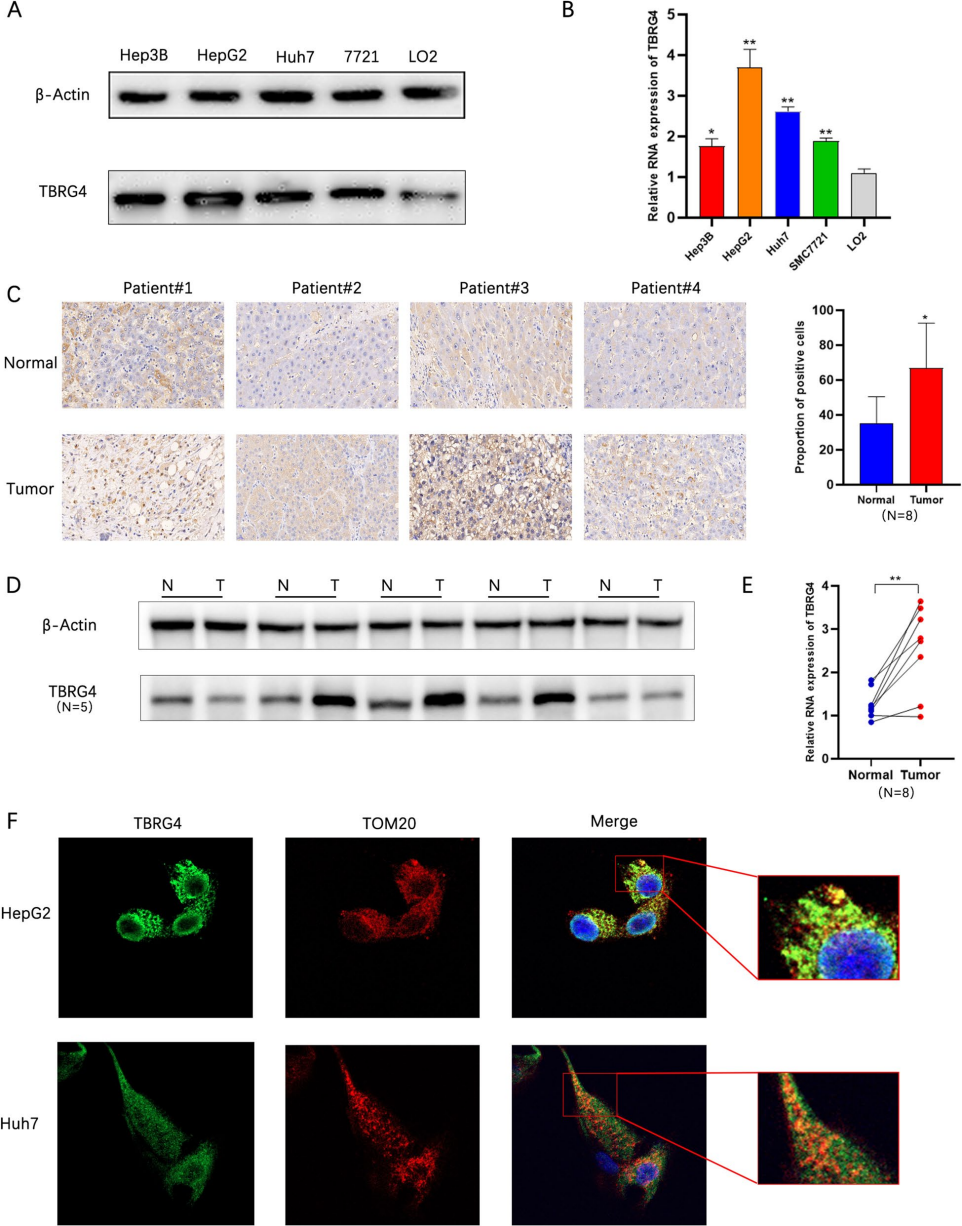
Verification of TBRG4 expression level. **A** Differences in protein expression levels of TBRG4 in HCC cell lines. **B** Differences in mRNA expression levels of TBRG4 in HCC cell lines. **C** Immunohistochemical staining of HCC cancer tissue and adjacent tissues (*n* = 8). Scale bars: 200 µm. **D** Differences in protein expression levels of TBRG4 in HCC cancer tissue and adjacent tissues (*n* = 5). **E** Differences in mRNA expression levels of TBRG4 in HCC cancer tissue and adjacent tissues (*n* = 8). **F** Immunofluorescence staining confirms the subcellular localization of TBRG4(Using FITC to label TBRG4 and CY3 to label mitochondrial protein TOM20). All the blots have been cropped and the original blot is shown in the Supplementary file and the samples derive from the same experiment and that gels/blots were processed in parallel. Data were shown as mean ± SD. *:* p* < 0.05, **: *p* < 0.01, ***: *p* < 0.001, ****: *p* < 0.0001

To further validate the expression of TBRG4 in HCC, we used Western blot and RT-PCR to detect the expression level of TBRG4 in liver cancer cell lines. The results showed that TBRG4 was significantly elevated in Hep3B, Huh7, SMC7721, and HepG2 tumor cells, while its expression was lower in human normal liver epithelial cells LO2 (Fig. 5A and B). In addition, we collected some HCC tumor tissues and adjacent tissues for immunohistochemical staining, and the results showed a significant increase in the proportion of TBRG4 positive cells in tumor tissues (Fig. 5C). Western blot and RT-PCR confirmed a significant increase in protein and mRNA levels of TBRG4 in tumor tissue (Fig. 5D and E). In addition, GO and KEGG enrichment analysis showed that TBRG4 is related to oxidative physiology and mitochondrial protein complex. Therefore, we used fluorescent antibodies to label mitochondria and TBRG4. We found that TBRG4 was localized on the mitochondria of HCC cells (Fig. 5F).

### Knocking down the expression of TBRG4 reduces the proliferation, migration, and invasion of HCC cells

**Fig. 6:**
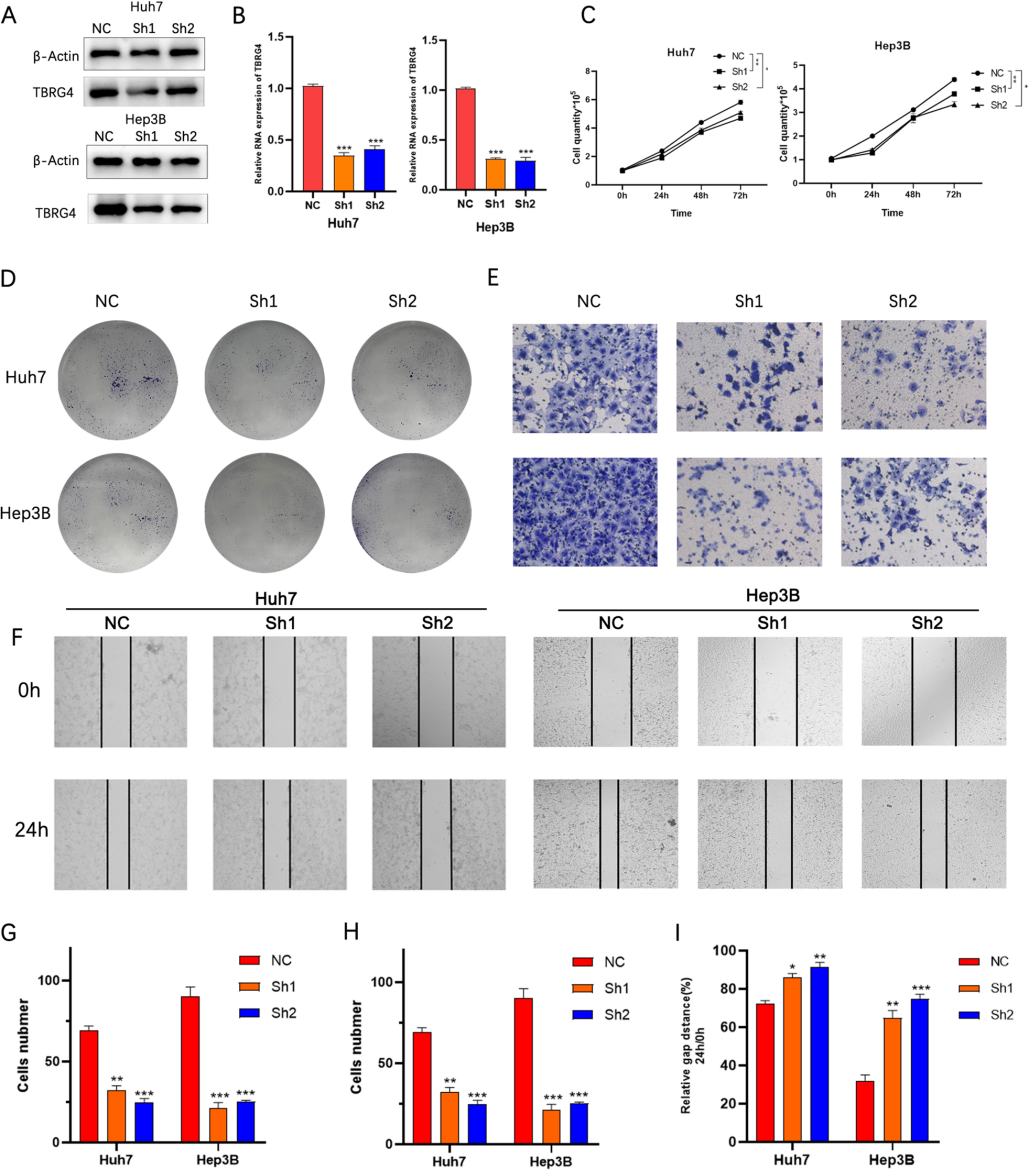
Biological function verification of TBRG4. **A** Differences in protein expression levels of TBRG4 after plasmid transfection into Hep3B and Huh7 cells. **B** Differences in mRNA expression levels of TBRG4 after plasmid transfection into Hep3B and Huh7 cells. **C** The proliferation of HCC cells was examined by cell counts. **D** Colony formation assay was employed to evaluate the proliferation of HCC cells. **E** Wound healing assay measured the motor ability of HCC cells. **F** The transwell assay detected the invasion of HCC cells. **G** The histogram represents the number of repeated bacteriolysis in each group based on colony formation measurements. **H** Histograms provide quantitative data on wound healing. **I** Differences in the number of cells passing through the chamber. All the blots have been cropped and the original blot is shown in the Supplementary file and the samples derive from the same experiment and that gels/blots were processed in parallel. Data were shown as mean ± SD. *:* p* < 0.05, **: *p* < 0.01, ***: *p* < 0.001, ****: *p* < 0.0001

To verify biological function of TBRG4 in HCC cells, we used interference plasmids to reduce the expression level of TBRG4. After 24 h of plasmid transfection, the protein and mRNA expression levels of TBRG4 showed significant downregulation (Fig. 6A and B). The growth curve and clone formation experiments indicate that knocking down the expression level of TBRG4 can significantly inhibit the proliferation and clone formation ability of HCC cells (Fig. 6C, D and G). In the traswell experiment, the TBRG4 knockdown group significantly reduced the number of invading cells (Fig. 6E, H and Supplemental Fig. 1G). Similarly, in the scratch experiment, the knocking out group of cells increased the width of wound healing (Fig. 6F and I). These results indicate that downregulating the expression level of TBRG4 can significantly inhibit the proliferation, migration, and invasion ability of HCC cells.

### TBRG4 regulates the proliferation and migration of HCC cells by DDX56/p-AKT/GSK3β signaling pathway

**Fig. 7:**
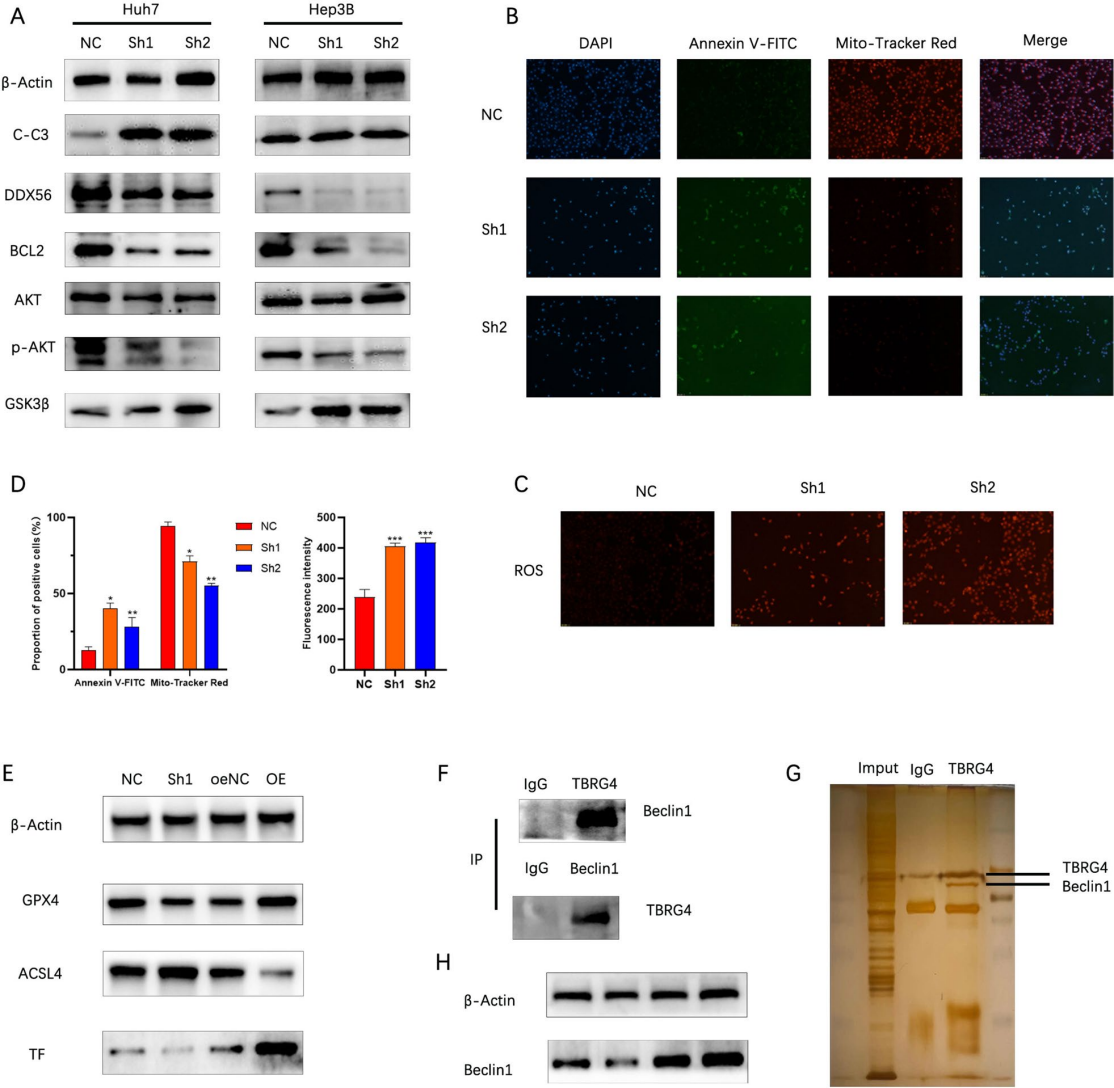
TBRG4 regulates the proliferation and migration of HCC cells by DDX56/p-AKT/GSK3β signaling pathway. **A** The expression levels of DDX56/p-AKT/GSK3β proteins and apoptosis-related proteins were evaluated by Western blot in control and TBRG4-konckdown cells. **B** Changes of mitochondrial membrane potential and apoptosis of HCC cells after TBRG4-knockdown (Annexin V was labeled with FITC, and mitochondrial membrane potential was labeled with Mito tracker red cmxros). **C** Changes of ROS in HCC cells after TBRG4-knockdown. **D** Statistical analysis of mitochondrial membrane potential, apoptosis ratio and ROS of HCC cells after TBRG4-knockdown. **E** Changes in ferroptosis -related protein markers after knockdown and overexpression of TBRG4. **F** Co-immunoprecipitation experiment showed the interaction between TBRG4 and Beclin1 in Huh7 cells. **G** Use agarose beads to capture the TBRG4 interaction protein, purify it, and analyze it on SDS-PAGE. Silver-stained gel shows different bands. **H** Changes in Beclin1 expression after knockdown and overexpression of TBRG4. All the blots have been cropped and the original blot is shown in the Supplementary file and the samples derive from the same experiment and that gels/blots were processed in parallel. Data were shown as mean ± SD. *:* p* < 0.05, **: *p* < 0.01, ***: *p* < 0.001, ****: *p* < 0.0001

To further explore the mechanism of TBRG4 involved in HCC progression, we used Western blot to detect signal molecules related to TBRG4. In the previous bioinformatics prediction, TBRG4 showed a strong correlation with DDX56. Studies have shown that DDX56 can inhibit the proliferation and migration of HCC cells through the PTEN/p-AKT signaling pathway [18]. We speculate that TBRG4 may participate in the AKT signaling pathway through DDX56. Western blot confirmed our hypothesis. We found that knocking down TBRG4 significantly decreased the expression of DDX56 and p-AKT, while the expression of Glycogen Synthase Kinase 3 Beta(GSK3β) significantly increased(Fig. 7A). This suggests that TBRG4 may participate in the proliferation and migration of tumor cells through the DDX56/p-AKT/GSK3β signaling pathway.

### Silencing TBRG4 can induce ferroptosis in HCC cells

Next, we examined the changes in HCC-related apoptotic proteins after silencing TBRG4.We found that B-cell lymphoma 2 (BCL2) significantly decreased, while Cleaved-Caspase 3 expression significantly increased. This indicates that silencing TBRG4 can induce programmed cell death in HCC cells (Fig. 7A). Therefore, we examined the mitochondrial membrane potential and apoptosis ratio of Huh7 cells. It was found that after silencing TBRG4, the mitochondrial membrane potential of Huh7 cells significantly decreased, and the proportion of apoptotic cells increased (Fig. 7B and D). ROS level detection showed that silencing TBRG4 significantly increased the ROS level in Huh7 cells (Fig. 7C and D). These results suggest that silencing TBRG4 may induce iron death in HCC cells. Therefore, we examined the changes in iron death-related markers in Huh7 cells. It was found that Glutathione peroxidase 4(GPX4) and Transferrine(TF) significantly decreased, while Acyl-CoA synthetase long-chain family member 4(ACSL4) significantly increased (Fig. 7E). To better understand the mechanism of TBRG4 regulating HCC ferroptosis, we verified the binding between ferroptosis-related proteins and TBRG4 through Co-IP. The reciprocal Co-IP experiment showed that TBRG4 can specifically bind to Beclin1 (Fig. 7F). Coomassie Brilliant Blue staining also confirmed our results (Fig. 7G). In addition, interfering with and overexpressing TBRG4 can regulate the expression level of Beclin1 (Fig. 7H). These results indicate that TBRG4 may regulate ferroptosis in HCC cells through its interaction with Beclin1.

## Discussion

Compared with other tumors, liver cancer has the characteristics of high invasiveness and easy recurrence and metastasis, and its high degree of malignancy is related to mitochondrial dysfunction [19]. Research has confirmed that mitochondria play an important role in the progression of liver cancer, and many aspects of mitochondrial energy production and even mitochondrial biology (including mitochondrial synthesis, kinetics, metabolism, signal transduction, cell death regulation, etc.) are related to liver cancer [20, 21]. In this study, it was determined that the mitochondrial metabolic gene TBRG4 serves as a significant promoter of hepatocellular carcinoma (HCC) proliferation and functions as an independent prognostic marker. Gene enrichment analysis revealed the involvement of TBRG4 in various mitochondrial functional pathways, such as oxidative phosphorylation and NADH dehydrogenase (ubiquinone) activity, as well as mitochondrial protein complex. Furthermore, TBRG4 was found to be associated with multiple signaling pathways related to tumor development, including Beta catenin, B cell receptor, and TP53 signaling pathway.

TBRG4 plays an important role in the progress of many cancers, including oral squamous cell carcinoma [22], lung cancer [23], osteosarcoma [24], and colorectal cancer [25]. However, the function of TBRG4 in HCC tumorigenesis has not yet been elucidated. The results of this study indicate that the expression level of TBRG4 increases in HCC tissues and cells. Suppressing the expression of TBRG4 can significantly inhibit the proliferation, migration, and invasion of HCC cells in vitro. These findings strongly suggest that TBRG4 plays a critical role in the progression of HCC, despite the limited information available on its exact mechanisms of action.

DDX56 is a component of 65S ribosomal precursor particles that regulate various RNA metabolism processes, including transcription [26]. Research has shown that DDX56 interacts with MECOM to promote the growth of HCC cells through the PTEN/p-AKT signaling pathway [18]. Our study found that downregulating TBRG4 can reduce the expression of DDX56/p-AKT. GSK3β is a multifunctional serine/threonine kinase and a downstream substrate and effector of AKT [27]. It is currently believed that GSK3β has been proven to regulate the protein hydrolysis and subcellular localization of cyclin D1, thereby affecting cell cycle and proliferation [28]. In addition, GSK3β can also affect the expression of EMT related genes, thereby regulating cell migration and invasion [29]. Our study found that downregulating TBRG4 can upregulate GSK3β through DDX56/p-AKT to inhibit the proliferation and migration of HCC cells. Therefore, the TBRG4/DDX56/p-AKT/GSK3β signal pathway may become a new therapeutic target for HCC.

Ferroptosis is a recently discovered iron-dependent programmed cell death. The metabolic characteristics and iron dependence exhibited by cancer cells make it possible to treat tumors with ferroptosis inducers, and promoting ferroptosis can effectively intervene in tumor progression [30, 31]. During ferroptosis, large accumulation of intracellular iron ions, mitochondrial dysfunction and the production of reactive oxygen species [32, 33]. Previous research has demonstrated that the inhibition or deficiency of GPX4 can directly result in the build-up of lipid peroxides and iron, ultimately leading to iron death in HCC cells [34]. ACSL4 plays a role in regulating the susceptibility of ferroptosis by primarily facilitating the metabolism of polyunsaturated fatty acids [35]. Our research revealed that reducing the expression level of TBRG4 results in a decrease in mitochondrial membrane potential, an increase in the proportion of apoptotic cells, and an accumulation of intracellular ROS. Western blot showed that downregulating TBRG4 can inhibit the expression of GPX4 and TF, and induce the expression of ACSL4, thereby promoting the lipid peroxidation of polyunsaturated fatty acids and inducing ferroptosis in HCC cells. By targeting TBRG4 with drugs, it is possible to enhance the ferroptosis of HCC cells through the GPX4/ACSL4 pathway, ultimately improving the prognosis of clinical patients.

Beclin1 is a key regulatory factor for autophagy and has been reported in the pathophysiology of metabolic disorders and tumors [36]. Ferroptosis is also an autophagy dependent cell death [37]. Research has shown that Beclin1 can form complexes with the core component SLC7A11 of System Xc-(cysteine glutamate reverse transporter), thereby promoting lipid peroxidation and iron death [38]. Inhibiting the expression of Beclin1 can reduce the occurrence of cell ferroptosis [39]. Our research has revealed that TBRG4 exhibits a specific binding affinity towards Beclin1. Furthermore, altering the expression level of TBRG4 has been observed to have a regulatory effect on the expression level of Beclin1. These findings strongly suggest a potential interaction between TBRG4 and Beclin1 in the joint regulation of ferroptosis.

In summary, we have successfully identified and validated a novel prognostic marker for hepatocellular carcinoma (HCC). Our findings demonstrate that TBRG4 exhibits abnormal elevation in HCC cells and is significantly associated with a poor prognosis. In vitro experiments have revealed that interference with TBRG4 can effectively inhibit the proliferation, migration, and invasion of HCC cells, while also inducing cell ferroptosis. Furthermore, our results suggest that TBRG4 may play a role in the proliferation and migration of HCC cells through the DDX56/p-AKT/GSK3β pathway and interact with Beclin1 to regulate cell ferroptosis. However, it is important to note that the limitation of this study lies in the need for a larger number of tissue samples to further validate the differential expression of TBRG4. Additionally, further investigation is required to elucidate the detailed mechanisms by which TBRG4 influences HCC progression.

### Supplementary Information

**Additional file 1: Supplemental Fig. 1.** Validation of TBRG4 in ICGC cohort and migration experiment. **Supplemental Fig. 2.** Original image of western blot experiment.

**Additional file 2: Supplemental Table 1. **The detailed information about the correlation between gene expression and drug sensitivity in pan-cancer.

## Data Availability

All data included in this study are available upon request by contact with the corresponding author. The datasets generated and during the current study are available in the TCGA (https://www.cancer.gov/ccg/research/genome-sequencing/tcga), Gene Expression Omnibus (https://www.ncbi.nlm.nih.gov/geo/) including GSE14520 and ICGC database (https://dcc.icgc.org/releases/current/Projects).

## Abbreviations

HCC: Hepatocellular carcinoma
TBRG4: Transforming growth factor beta regulator 4
DDX56: DEAD-Box 56
GSK3β: Glycogen Synthase Kinase 3 Beta
BCL2: B-cell lymphoma 2
GPX4: Glutathione peroxidase 4
ACSL4: Acyl-CoA synthetase long-chain family member 4
TF: Transferrin
