# Supplementary material for: High expression of TBRG4 in relation to unfavorable outcome and cell ferroptosis in hepatocellular carcinoma
Source: BMC Cancer. 2024 Feb 12;24:194. doi: 10.1186/s12885-024-11943-1 (PMC10860303; doi:10.1186/s12885-024-11943-1)

Supplemental figure1: Validation of TBRG4 in ICGC cohort and migration experiment


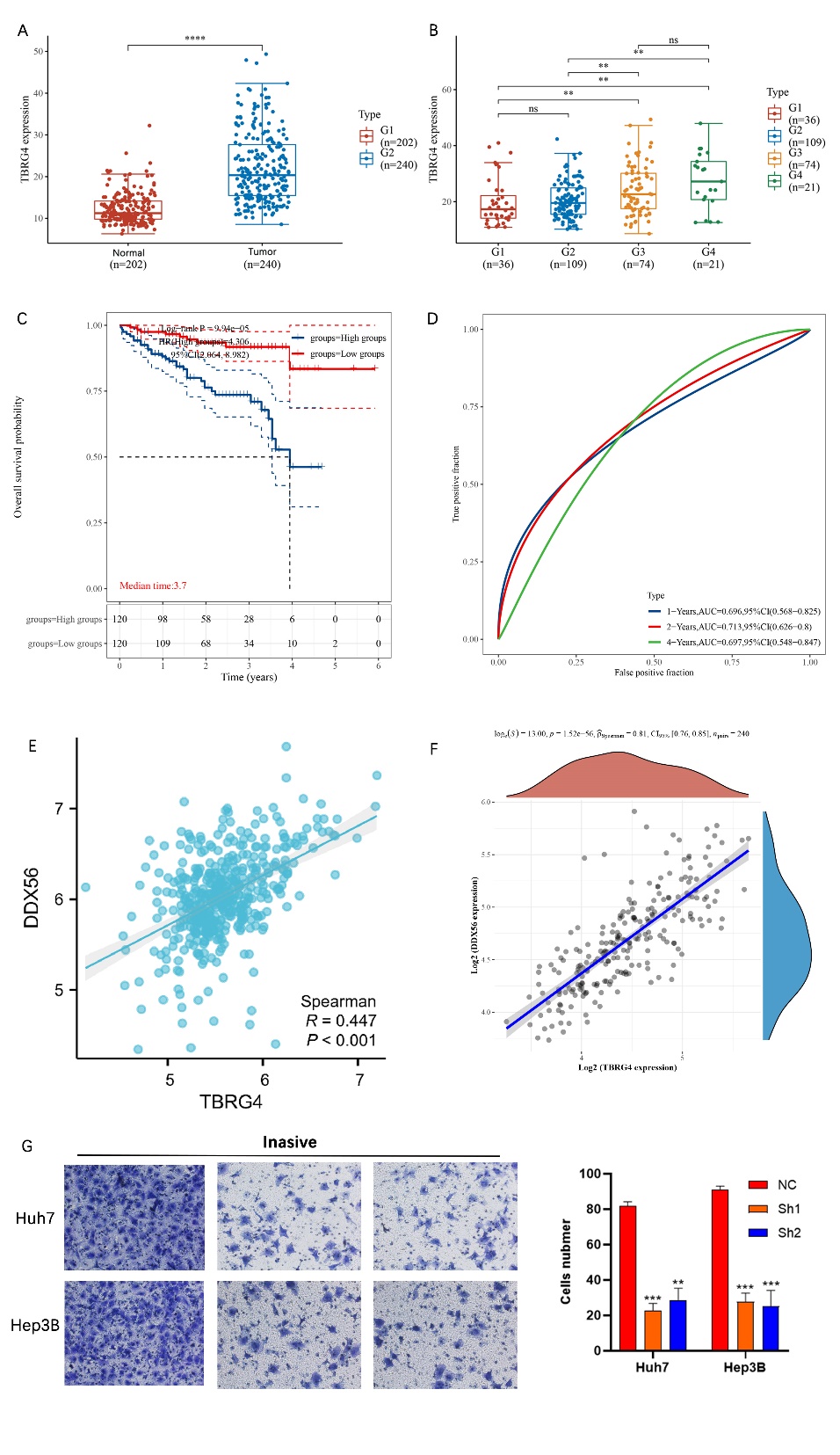


A: Differential expression of TBRG4 in the ICGC cohort. B: Differential expression of TBRG4 in pathological grades of ICGC patients. C: Analysis of the correlation between TBRG4 expression and ICGC patient OS. D: Analysis of predictive efficacy of TBRG4 for OS of ICGC patients. E: Correlation analysis of TBRG4 and DDX56 expression in GSE14520. F: Correlation analysis of TBRG4 and DDX56 expression in ICGC. G: The transwell assay detected the migration of HCC cells. Data were shown as mean ± SD. *: p < 0.05, **: p < 0.01, ***: p < 0.001, ****: p < 0.0001.

Supplemental figure2: Original image of western blot experiment


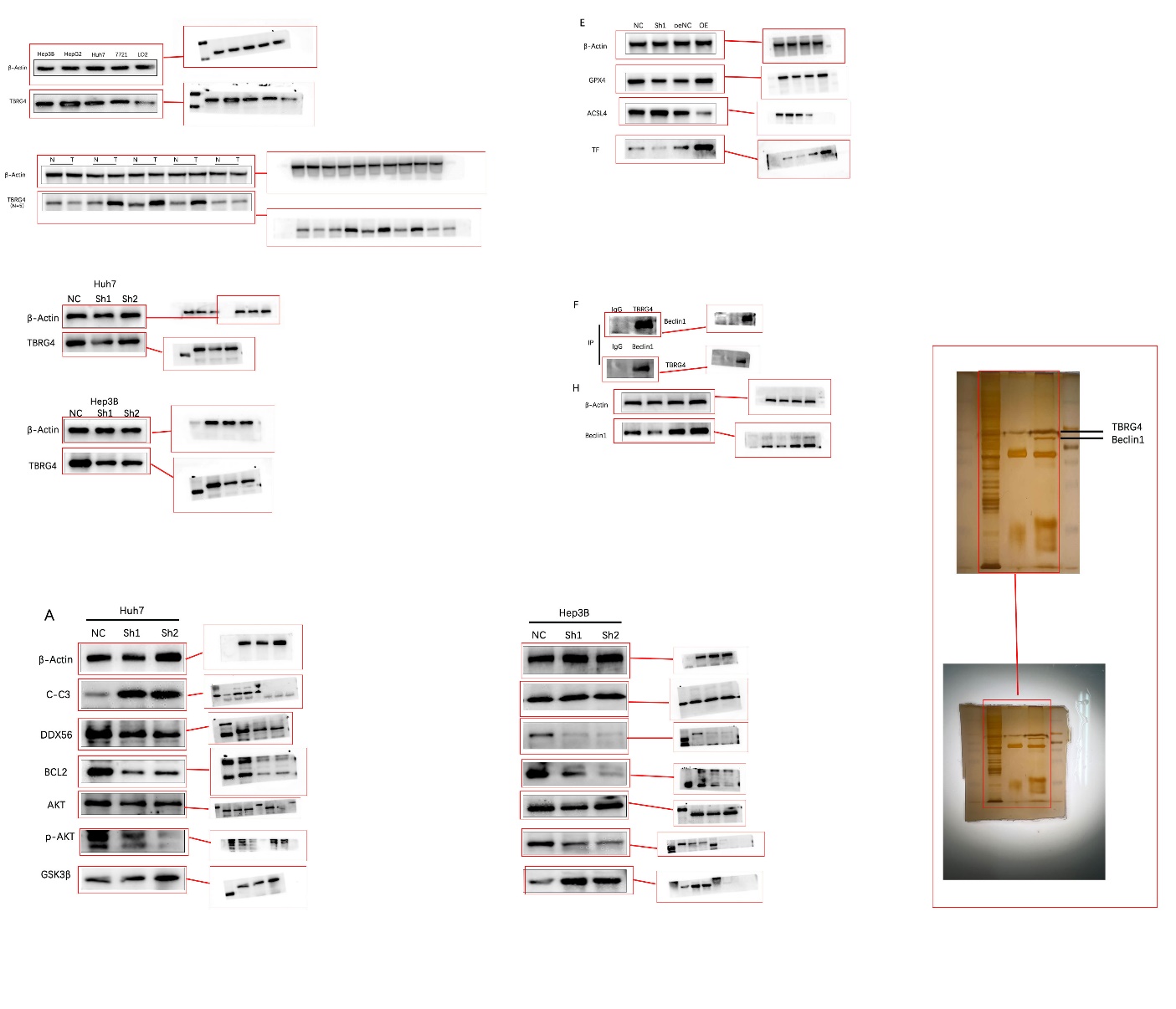

Supplement: Supplementary file 1 — Additional file 1: Supplemental Fig. 1. Validation of TBRG4 in ICGC cohort and migration experiment. Supplemental Fig. 2. Original image of western blot experiment. [file 12885_2024_11943_MOESM1_ESM.docx]
